# Are we missing the mark? Relationships of psychosocial issues to outcomes after injury: A review of OTA annual meeting presentations

**DOI:** 10.1097/OI9.0000000000000070

**Published:** 2020-04-23

**Authors:** Natasha M. Simske, Mary A. Breslin, Sarah B. Hendrickson, Heather A. Vallier

**Affiliations:** MetroHealth Medical Center, Cleveland, Ohio, affiliated with Case Western Reserve University

**Keywords:** injury, mental illness, outcomes, pain, PROM, substance abuse, trauma

## Abstract

**Objectives:**

To observe the availability of information about social, emotional, and psychological factors in abstracts presented at the Orthopaedic Trauma Association (OTA) annual meeting.

**Data source:**

OTA website (https://ota.org/education/meetings-and-courses/meeting-archive/)

**Study Selection:**

All abstracts selected for paper or poster presentation at the 2016 through 2018 OTA annual meetings, as published in the final program. Studies were included if they sought to measure mental illness, substance use or abuse, pain, or other psychosocial issues. If studies utilized 1 or more patient-reported outcome measures (PROMs), they were also included.

**Data extraction:**

For each abstract meeting inclusion criterion, studies were assessed for interventions intended to improve outcomes in any of the listed psychosocial domains.

**Data synthesis/Results:**

Nine hundred forty-two abstracts were evaluated over a 3-year period. Of these, 294 (31.2%) met inclusion criteria. Twenty-five abstracts (8.5% of 294) reported mental illness, with depression (n = 14), anxiety (n = 9), and posttraumatic stress disorder (n = 5) being the most common. Eighty-eight abstracts (29.9% of 294) reported substance-use of tobacco, alcohol, narcotics, and/or recreational drugs. Tobacco-use was most prevalent (n = 59), followed by opioid-use (n = 31). Ten abstracts reported substance abuse. Pain was measured in 95 abstracts, and 203 abstracts utilized PROMs. Thirty-five abstracts found that these psychosocial elements significantly impacted outcomes or complications. Many abstracts did not assess the influence of these factors on clinical outcomes (n = 99). Sixteen studies described an intervention aimed at mitigating these features.

**Conclusions:**

This study illustrates limited attention to the impact of psychological, social, and environmental factors on outcomes after orthopaedic trauma. Substance-abuse problems and mental health concerns are not only predictors of poor clinical and PROMs of pain and quality of life after injury, but have also been implicated in subsequent recidivism. Only 3% of 942 abstracts observed mental health and 1% reported substance-abuse. Moving forward, greater understanding of psychosocial issues may enhance interventions to impact long-term outcomes.

## 1 Introduction

The effects of traumatic injury often persist following hospital discharge. As a result, clinicians have become increasingly aware of the importance of addressing psychosocial concerns including mental illness, substance-use, pain, social support, and self-efficacy after injury. Psychiatric illness and substance-use disorders are a leading cause of disability in the United States and worldwide.^[1,2]^ In trauma patient populations, rates of psychiatric illnesses have reportedly reached as high as 45%.^[3–10]^ Substance use is likewise more prominent in trauma populations.^[11,12]^ Not only have mental health disorders been linked to higher rates of complications, worse outcomes, and poor adherence, but such patients are also at higher risk for subsequent recidivism.^[4,6,9,10,13–19]^

Postoperative pain, narcotic use, and opioid prescription practices following orthopaedic injury have been well explored. Since opioid use has been linked with a number of adverse side effects and places patients at risk for overuse and addiction, some studies have sought to evaluate innovative means of reducing opioid use after traumatic injury.^[20–24]^ Evidence indicates that psychiatric illness, particularly depression and anxiety, can alter pain perception and therefore may play an essential role in this relationship^[3,7,9,25]^

Interventions to address psychosocial issues in this population have been limited, despite contributions made by large, collaborative research groups including the Lower Extremity Assessment Project and the Major Extremity Trauma Research Consortium, through the Trauma Collaborative Care Study.^[26–29]^ Counseling, education, and other interventions have proven beneficial in limiting opioid use^[30]^ and in bolstering access to mental health resources.^[31,27,32]^ The purposes of this study were to describe the frequency of information about social history and psychiatric health in abstracts presented at the OTA annual meeting and to identify opportunities for future study and intervention.

## 2 Patients and methods

Abstracts included in the final program at OTA annual meetings from 2016 through 2018 were retrospectively reviewed using the publicly assessable meeting archives: https://ota.org/education/meetings-and-courses/meeting-archive/. Both paper and poster presentation abstracts were included. A researcher not involved in clinical care read each of the 942 applicable abstracts for inclusion. The inclusion criterion was utilization or analysis of a psychosocial factor, namely mental illness, substance use or abuse, pain, or other concerns (e.g., satisfaction, self-efficacy, or social support). Abstracts were also included if they incorporated scores from patient-reported outcome measures (PROMs) in their methodology or results.

Each abstract was also analyzed to determine if the psychosocial variable (e.g., depression) was associated with clinical or functional outcomes (e.g., depression correlated with more revision surgeries). Abstracts that only reported functional outcomes through use of 1 or more PROMs were excluded from this analysis, as it was deemed not applicable. Presence of an intervention to address 1 or more of these issues was also recorded.

Descriptive, univariate analyses were used to describe mental illnesses, substance use, and functional outcome questionnaires. To assess for any potential changes made over time, categorical variables in abstracts from 2016, 2017, and 2018 were compared using Chi-squared tests. In all cases, *P* < .05 indicated a statistically significant difference between groups.

## 3 Results

### 3.1 Mental illness

**Figure 1:**
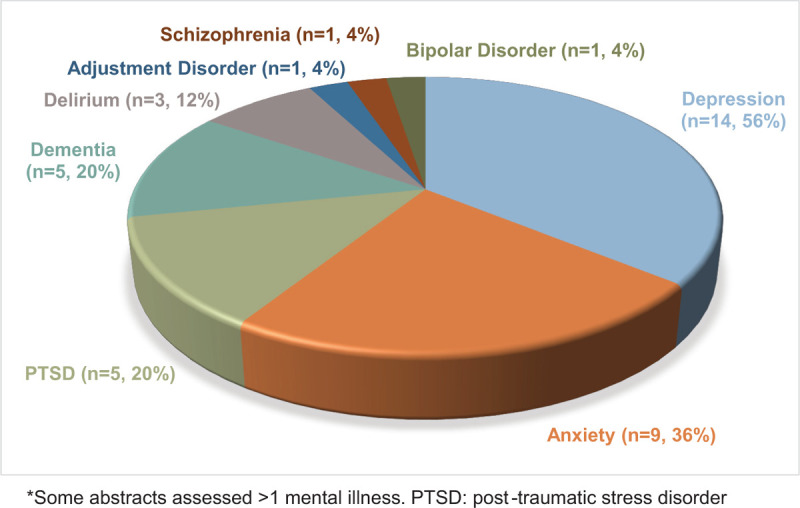
Distribution of psychiatric disorders among abstracts reporting mental illness (n = 27)∗.

Nine-hundred forty-two abstracts were assessed from 3 OTA annual meetings. Of these, 294 abstracts (31.2%) met inclusion criteria. Three percent of abstracts assessed mental illness (27 of 942). The 2018 meeting had the most abstracts that assessed or were inclusive to psychiatric illness (4% vs 2% in both 2016 and 2017) (Table 1). In these applicable abstracts, depression was the most common (n = 14), followed by anxiety (n = 9) and posttraumatic stress disorder (n = 9) (Fig. 1).

**Table 1:**
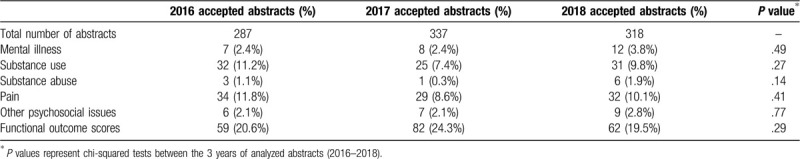
Report of abstracts that included some form of analysis or observation of psychosocial factors (n = 294 of 942, 31%).

### 3.2 Substance-use and other psychosocial factors

**Figure 2:**
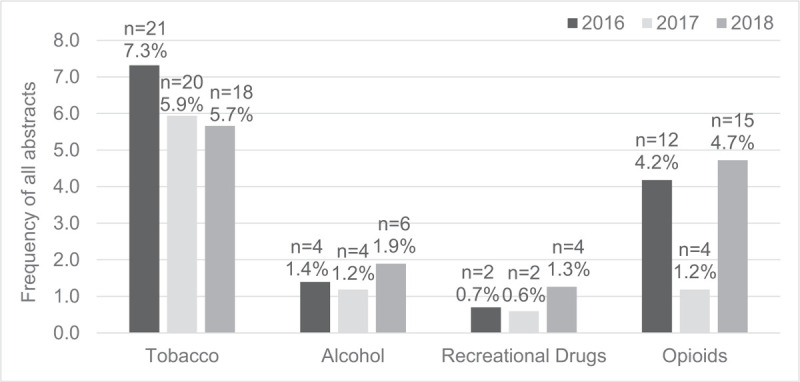
Breakdown of reported substances among all abstracts (n = 942).

Reporting substance-use was more common, with 88 of 942 abstracts (9.3%), while only 10 abstracts (1.1%) measured substance abuse (Table 1). Tobacco use or smoking history was most frequently reported, with 5% to 7% of abstracts including this variable in analyses from 2016 to 2018 (Fig. 2). Opioid use was also common (31 abstracts, 3.2%). Alcohol use and recreational drug use, including marijuana, were not as common. Pain was evaluated in 95 abstracts (9.8%), typically using a visual analog scale. Other psychosocial issues were measured in 21 of the 942 abstracts (2.2%). These included resilience, social support, self-efficacy, coping, catastrophizing, and satisfaction, among other factors.

### 3.3 Functional outcomes

Functional outcome scores were used in 203 of the 942 abstracts (21.5%) and 340 surveys were used (average: 1.7 per abstract) (Table 2). Disabilities of the Arm, Shoulder, and Hand were most common (n = 51, 25% of the 203), followed by the Short form-12 or 36 (n = 44, 22%), and the Musculoskeletal Function Assessment (MFA; n = 42, 21%). Fifty-five distinct measures were used, with the majority looking at limb or joint-specific outcomes. See Table 2 for greater detail.

**Table 2:**
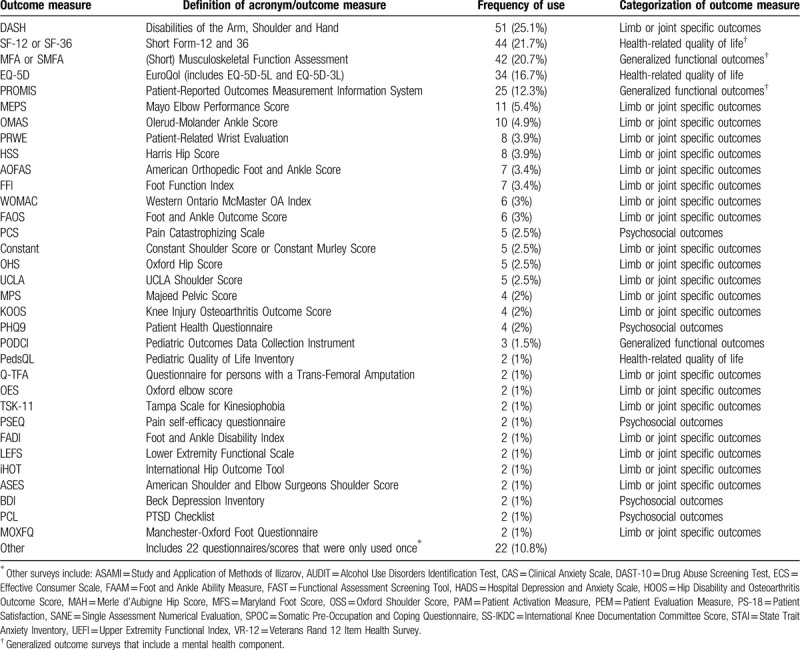
Description of patient-reported outcome measures (PROMs) used in the N = 203 applicable abstracts.

### 3.4 Effect on outcomes and use of interventions

Thirty-seven abstracts (12.6% of 294) found that these psychosocial factors had a significant (negative) impact on results or outcomes, such as complication rates or functional outcome scores (Table 3). Contrary to this, 32 abstracts (10.9%) did not identify a meaningful impact of psychosocial factors on outcomes. However, many studies did not assess the relationship between such factors and outcomes (n = 103, 35.0%). Over the 3-year period, 16 studies (5.4% of 294) described an intervention aimed at mitigating the impact of these factors. Ten of 16 interventions (62.5%) pertained to pain following injury, 4 (25%) regarded opioid use and the others addressed tobacco use, mental health or satisfaction. Two of these interventions pertained to both pain levels and opioid use.

**Table 3:**
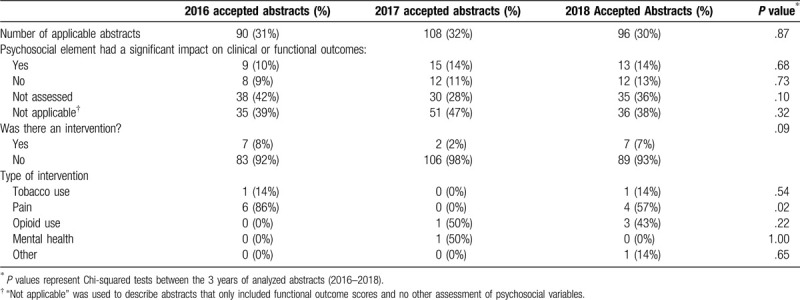
Assessment of impact of psychosocial variables on outcomes and use of interventions in applicable abstracts (n = 294 of 942).

## 4 Discussion

This study illustrates limited prior study of psychological, social, and environmental features on recovery following traumatic orthopaedic injury. A limited number of abstracts (31%) met inclusion criteria and 41% of these abstracts only used 1 or more patient-reported functional outcome scores. Very few abstracts reported psychiatric illness (3%) or substance abuse (1%). This analysis indicates potentially unmet needs or potential outcome effects among this population and an opportune area of broad study and impact.

Approximately 10% of all abstracts (95 of 942) evaluated pain following injury. Out of all psychosocial variables assessed, pain was the most prevalent. Pain and associated medication use and prescription practices are well assessed in more recent orthopaedic literature. Ongoing investigation is still crucial to determine patients at risk for chronic pain and to mitigate associated poor outcomes and potential overuse of pain medication. Pain following orthopaedic trauma is linked with multiple psychosocial phenomenon, including but not limited to long-term disability, failure to return to work or major daily activities, and decreased satisfaction.^[28,33,34]^ Studies have sought to understand opioid prescription and usage practices, as well as factors predicting overuse or noncompliance with prescribed pain medication, some of which are modifiable, social factors.^[35,36]^ Ten of 16 interventions over the 3-year period sought to improve pain after orthopaedic injury, indicating that we are accurately addressing these issues.

Substance use was assessed frequently, with 9% of abstracts observing patient use of tobacco, alcohol, recreational drugs, or opioids. Substance use is more common in trauma populations and has long been identified as a major risk factor for traumatic injury and more severe injuries.^[11,12,37,38]^ Despite some abstracts reporting substance use (9%), very few abstracts described interventions to mitigate tobacco use (2 abstracts) or opioid use (4 abstracts). No studies attempted to curtail alcohol or recreational drug use. In a large survey of over 800 orthopaedic trauma patients, McCrabb et al^[39]^ found that despite > 75% of current smokers being somewhat or very interested in quitting smoking, less than half received advice from their surgeon to quit smoking during their hospital stay. This points to a paucity of smoking cessation interventions that are welcomed by a majority of tobacco-using orthopaedic patients.

Only 3% of the 942 total abstracts reported on or measured psychiatric illness, despite high rates (up to 45%) of psychiatric illness in trauma populations.^[3–9]^ Depression and anxiety were mentioned most often, consistent with the reported occurrence of these disorders in trauma populations.^[9]^ Thirteen of 27 abstracts assessed whether mental illness significantly influenced outcomes. Of these, 7 of 13 (54%) observed substantial negative effects, including more complications, worse physical function, and greater resource utilization. These results are in keeping with existing evidence indicating that psychiatric illness is both associated with and a predictor of complications, lower satisfaction, and worse functional outcomes following orthopaedic injury.^[6,8,9,17–19,28]^

Some studies have highlighted the complex relationship between chronic pain and mental illness. Pain is common after orthopaedic injury and operative treatment, can foster anxiety, catastrophizing, and new or worsening mental illness.^[40,41]^ In turn, psychiatric illness predominates in populations with chronic pain, though the causal relationship between the two is not necessarily clear.^[42,43]^ Comorbidity of chronic pain and depression is associated with worse outcomes, such as reduced function and poor treatment response, compared with situations where only 1 condition is present.^[44,45]^ Ten abstracts (1% of 942) measured some form of psychiatric illness and pain. Future in-depth investigation of these frequently intertwined psychosocial hurdles is pertinent.

The strength of this paper is the attention to a topic that has been minimally addressed in prior trauma literature, and one that appears to play a critical role in recovery. However, due to the retrospective nature of this analysis, the authors cannot be sure that these psychosocial phenomena were not touched upon in the full presentation format (whether paper or poster). The authors posit, however, that if these variables or factors were a prominent component of the study, they should be mentioned in the abstract. This study is limited by the subjective nature of analysis as well. Only 1 researcher assessed the abstracts for inclusion and therefore interobserver reliability cannot be determined. Finally, only accepted abstracts were assessed, and therefore the authors cannot speak to the submitted studies that may have touched upon these topics but were not selected for poster or paper presentation.

Topics at the OTA annual meeting covers a wide range of basic and clinical topics, many of which would not be expected to include measurements of social, psychological, and functional aspects of recovery. Nevertheless, according to major research collaborations, investigation of psychological, social, and environmental subject matter remains limited in orthopaedic trauma literature. This is despite recent association of self-efficacy, social support, pain, substance abuse, and other mental illness with outcomes after trauma.^[26–29]^ This trend is accurately portrayed by the low representation of these topics at the OTA annual meeting over the past 3 years. Of a variety of psychosocial features assessed including mental illness, substance use and pain, the latter 2 were substantially more common (included in 9% and 10% of abstracts, respectively). Psychiatric illness and “other” psychosocial elements such as self-efficacy, social support, coping, and resilience were far less common. Given the subsequent publication rate for 66% of OTA presentations, there exists great potential for future high-quality studies to incorporate psychosocial data to promote extensive impact in these domains that broadly affect patients after traumatic injury.^[46]^
